# The accuracy of clinical diagnosis of onychomycosis in Dutch general practice: a diagnostic accuracy study

**DOI:** 10.3399/BJGPO.2022.0186

**Published:** 2023-06-28

**Authors:** Roeland M Watjer, Tobias N Bonten, Maikel AHM Arkesteijn, Koen D Quint, Martha T van der Beek, Liesbeth MH van der Raaij-Helmer, Mattijs E Numans, Just AH Eekhof

**Affiliations:** 1 Department of Public Health and Primary Care, Leiden University Medical Center (LUMC), Leiden, The Netherlands; 2 Department of Dermatology, Leiden University Medical Center (LUMC), Leiden, The Netherlands; 3 Department of Dermatology, Roosevelt Clinic, Leiden, The Netherlands; 4 Department of Medical Microbiology, Leiden University Medical Center (LUMC), Leiden, The Netherlands

**Keywords:** onychomycosis, diagnosis, microbiology, general practice, primary healthcare

## Abstract

**Background:**

Onychomycosis, the most common cause of nail dystrophy, is generally diagnosed by clinical examination. Current guidelines for Dutch general practice advise confirmatory testing only in cases of doubt or insufficient response to treatment. However, making a correct diagnosis can be challenging given the wide variety of clinical features and differential diagnosis.

**Aim:**

To establish accuracy of clinical diagnosis of onychomycosis by GPs.

**Design & setting:**

A diagnostic accuracy study based on GPs' clinical diagnosis of primary care patients suspected of onychomycosis.

**Method:**

Using 137 complete datasets from the Onycho Trial, diagnostic accuracy of clinical diagnosis as the index test was compared with confirmatory testing as the reference test. A sensitivity analysis was performed to determine diagnostic values for different combinations of index and reference test. Logistical regression was used to assess which clinical characteristics were associated with the positive predictive value (PPV) of the index test.

**Results:**

Clinical accuracy, that is the PPV of the index test, was 74.5%. Sensitivity analysis showed no significant difference in diagnostic values. Male sex and a history of any previous treatment significantly increased clinical accuracy with an odds ratio (OR) of 3.873 (95% confidence interval [CI] = 1.230 to 12.195, *P* = 0.021) and OR 4.022 (95% CI = 1.075 to 15.040, *P* = 0.039), respectively.

**Conclusion:**

The study demonstrated that the GPs' clinical diagnosis of onychomycosis was insufficiently accurate to initiate treatment without confirmatory testing. Further research is needed to investigate how to increase clinical accuracy and reduce potentially unnecessary exposure to treatment.

## How this fits in

Onychomycosis is often diagnosed by physical examination, but making a correct clinical diagnosis can be challenging owing to the broad differential diagnosis and wide variety of possible clinical features at presentation. Given the high prevalence of onychomycosis, it is important to establish the accuracy of clinical diagnosis of GPs, especially when treatment is considered. This study evaluated the accuracy of clinical diagnosis of onychomycosis in primary care by comparing the clinical diagnosis as the index test with confirmatory test results as the reference test. It found a significant chance of an incorrect clinical diagnosis without confirmatory testing; this was lower in males and those with a history of any previous treatment. These results should be taken into account when considering treatment and confirmatory testing in general practice.

## Introduction

Onychomycosis is a very common nail problem. It accounts for more than half of all nail-related disease and around 30% of fungal skin infections in Europe and North America.^1–3^ On average 4.3% of adults are affected, although rates vary between 2% and 14%, increasing substantially with increasing age.^4–7^ Onychomycosis can lead to discolouration, thickening, and separation from the nail bed; the great toenail is most often affected.^8,9^ The chance of a self-limiting course is very low.^10^

In general, onychomycosis has an indolent disease course, although some patients experience pain and discomfort through thickening of the nail and may develop complications such as recurring dermatomycosis or secondary bacterial infection.^9^ Additionally, several studies have shown significant decrease in nail-related quality of life in affected individuals.^11–15^

According to a Cochrane review, the most effective treatment is oral terbinafine or an imidazole for 3 months.^16^ However, oral treatment may have potentially serious side effects including severe skin rash and liver injury.^17^ Weighing potential adverse reactions against onychomycosis’ indolent disease course, physicians might be reluctant in prescribing oral treatment especially for milder cases.

Onychomycosis is often diagnosed on clinical examination. Accuracy of clinical diagnosis varies between different specialists. Dermatologists’ clinical diagnosis was confirmed by laboratory testing in 93% of cases, compared with 81% for GPs.^18^ Another study found podiatrists to be superior in clinically diagnosing onychomycosis with an accuracy of 80.8%, compared with 75.3% for dermatologists and 66.2% for GPs.^19^

In current Dutch general practice guidelines, antifungal treatment may be started solely based on clinical examination. Confirmatory testing by potassium hydroxide (KOH) preparation, fungal culture, or polymerase chain reaction (PCR) for fungal DNA is advised only when in doubt or in the case of treatment failure.^10^ These tests also have varying accuracies and generally modest sensitivity. KOH is the most sensitive (85%), followed by PCR (73%) and culture, which is the least sensitive (54%).^20,21^ To increase diagnostic accuracy, it is recommended to use a combination of different confirmatory tests.^21–23^

Given the varying accuracies of tests, patients could be unnecessarily exposed to treatment and its potential side effects. Confirmatory testing of clinically suspected cases could reduce potential overtreatment. To make a decision whether or not to use confirmatory testing in general practice, one needs to know the accuracy of clinical diagnosis.

This study aimed to determine the accuracy of clinical diagnosis of onychomycosis by GPs compared with confirmatory testing. Second, the study aimed to assess which clinical features have a significant impact on predicting confirmatory testing outcome.

## Method

### Study design

The accuracy of clinical diagnosis was investigated using data from the Onycho Trial, a randomised double-blind placebo-controlled trial (RCT), investigating the effectiveness of topical treatment with miconazole (Daktarin) or amorolfine (Loceryl) compared with placebo for mild to moderately severe onychomycosis in Dutch general practice (https://trialsearch.who.int/Trial2.aspx?TrialID=NL8193). Clinical diagnosis by the GPs involved was determined by the index test; confirmatory testing of the affected nail samples was used as the reference standard.

Regarding the index test, the conclusion of three independent observers was used based on standardised photographs taken during screening for the Onycho Trial, and these were compared with results of confirmatory testing. A positive clinical diagnosis by at least two out of three observers was regarded an overall positive clinical diagnosis; only one or no observer making a positive diagnosis was regarded as a negative clinical diagnosis. The three observers were two senior GPs (JE and TN) and one GP in training (RW). All observers were blinded for treatment allocation, clinical information recorded, and each other’s assessments.

For confirmatory testing, a positive result from any one of three testing methods used (KOH, PCR, or culture) was considered a positive reference test. PCR consisted of lab developed real-time PCR assays designed to detect trichophyton and microsporum species, based on the assay described by Wisselink *et al* and an assay to detect *Trichophyton rubrum*.^24^ Note that not all participants received all three types of confirmatory tests. All patients did receive KOH and additional PCR testing, but fungal culture was only performed for determination purposes in case of a positive KOH with negative PCR results and in case of a positive dermatophyte PCR with a negative *T. rubrum* species PCR. This was in accordance with standard work-up protocols of the affiliated departments of dermatology and microbiology.

Finally, the clinical diagnosis was considered to be correct if the overall clinical diagnosis made by the observers was in agreement with the overall confirmatory test result.

### Patients

All study participants (aged 18–70 years) with mild onychomycosis recruited and screened for the Onycho Trial, between October 2019 and January 2022, were considered for analysis. The majority of patients were recruited from the general public through social media; others were directly or indirectly referred by GPs affiliated with the regional research collaboration ‘Extramural Leiden Academic Network’ (ELAN).

### Data collection

All data originated from the ongoing Onycho Trial. Castor Electronic Data Capture (EDC), a cloud-based data management system, was used to record all data. Only datasets consisting of complete data, on both clinical diagnosis and laboratory test results, were used for the analyses.

### Statistical analysis

Diagnostic values, that is, sensitivity, specificity, negative predictive value (NPV), and positive predictive value (PPV) representing the accuracy of the clinical diagnosis, were calculated using 2 × 2 tables, with clinical diagnosis as the index test and the overall result of confirmatory testing as the reference test. To compare observers, diagnostic values were calculated for each observer separately. For the level of agreement between observers, descriptive statistics and inter-observer reliability (Fleiss' kappa) were calculated.

To evaluate differences between possible combinations of index and reference test and to compare these to the primary combination chosen, a sensitivity analysis was performed constructing 2 × 2 tables for all possible combinations between number of observers making a positive clinical diagnosis (1 out of 3 up to 3 out of 3) and all possible combinations for confirmatory testing. Diagnostic values were calculated for each combination and their 95% CI using Wilson’s score method for binomial proportions.

Uni- and multi-variate logistic regression analyses (forced entry and stepwise methods) were performed to assess whether any of the patient characteristics were significantly associated with the accuracy of clinical diagnosis, calculating ORs and their 95% CIs. For categorical data, the lowest or least severe category was chosen as the reference group.

Finally, a subgroup analysis was performed for the significantly associated characteristics found by multivariate regression. For all analyses performed, a *P* value of <0.05 was considered statistically significant.

Statistical analyses for both sensitivity and regression analyses were conducted using IBM SPSS (version 25).

### Sample size

To calculate the required sample size for the binary outcome, that is, having onychomycosis or not, the following equation was used: *n* = 2 (zα_⁄2_ + zβ)^2^p̅(1 − p̅)/d^2^, with an alpha of 0.05 and a power of 90%. Setting the PPV of combined confirmatory testing at 100% and the estimated accuracy of clinical diagnosis of GPs at 70%, which was based on available literature, the expected difference was 30%.^19^ Using these numbers, a minimum of 30 was required for both groups resulting in a total sample size of 60 patients.

## Results

### Patient selection

**Figure 1.**
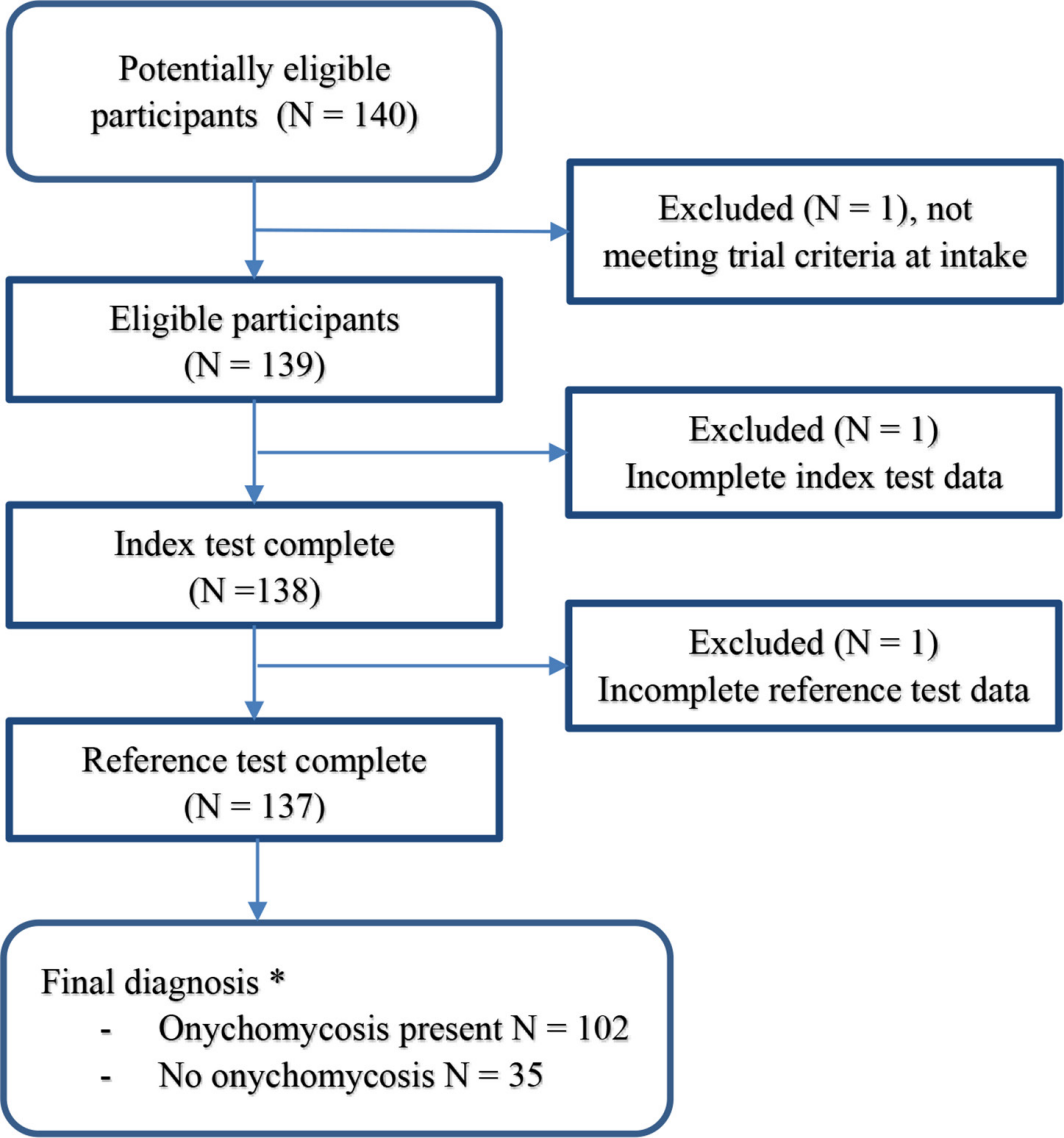
Participant flow diagram. *Confirmed by at least 2 out of 3 observers

Of 140 available records, 137 had complete data necessary for analyses. For the other three not all observers had provided the required input, or results of confirmatory testing were still pending or not performed (Figure 1). Regarding the incomplete records, the first patient was excluded at intake owing to metformin use, not collecting any further data. The second did not receive a clinical diagnosis from all three observers as required for analyses. For the third patient, no PCR test was performed owing to shortages caused by the COVID-19 pandemic, required for the reference standard (Figure 1). Since the reasons for these incomplete observations were unrelated to the potential value of the outcome, these data were considered to be missing at random; that is, not causing bias.

### Patient characteristics

Table 1 shows the study population’s characteristics and risk factors for onychomycosis. The majority (59.1%) of the patients were aged >50 years and had moderate-to-severe onychomycosis (based on Onychomycosis Severity Index^25^ of 3–4 toenails and moderate symptoms for 1 year). The vast majority (83.2%) had tried any form of treatment including oral antifungal medication, to which only 17 patients (12.4%) had been exposed explicitly. All previous treatments, including home remedies, were self-reported; no data were available to verify type, timing, or duration of any treatment.

**Table 1.** Participant characteristics as frequency (*n*) and proportion (%), unless otherwise stated

Demographic factors	Total(*n* = 137)
**Mean age, years (SD)**	52.0 (12.5)
0–25 years	4 (2.9)
26–50 years	52 (38.0)
51–75 years	81 (59.1)
**Male**	60 (43.8)
**Skin type (Fitzpatrick skin types)**	
Very fair, fair, and medium	133 (97.1)
Olive, brown, and black	4 (2.9)
**Clinical factors**	
**Affected area of index toenail**	
1–10%	6 (4.4)
11–25%	31 (22.6)
26–50%	57 (41.6)
51–75%	34 (24.8)
76–100%	9 (6.6)
**OSI score**	
Mild (0–5)	20 (14.6)
Moderate (6–15)	70 (51.1)
Severe (16–35)	47 (34.3)
**Mean total affected toenails (SD)**	3.8 (1.9)
**Time since onset ≥1 year**	118 (86.1)
**Score ONYCHO questionnaire**	
Good (67–100%)	47 (34.3)
Moderate (34–66%)	83 (60.6)
Poor (0–33%)	7 (5.1)
**Risk factors**	
History tinea pedis	78 (56.9)
Family members with onychomycosis	31 (22.6)
Presence of dermatological disease	40 (29.2)
History of oral treatment	23 (16.8)
History of any previous treatment	114 (83.2)
History of use of immunosuppressives	17 (12.4)
Frequent use of public pool or spa (≥5 visits/year)	49 (35.8)
Consistent use of flip-flops during these visits	32 (23.4)
History of smoking or actively smokes	57 (41.6)

OSI = Onychomycosis Severity Index.

### Accuracy of clinical diagnosis

Of 137 patients included, 102 were correctly diagnosed, that is, confirmed by laboratory testing; 35 were incorrectly diagnosed (Table 2). Clinical accuracy, that is, the PPV, was 74.5% (95% CI = 66.2% to 81.3%). Comparing clinical accuracy, there was no significant difference between the different observers (Table S1). Regarding inter-observer agreement, there was unanimous agreement on a positive clinical diagnosis in 127 of 137 cases (92.7%). However, for the remaining cases where at least one observer considered an alternative diagnosis, in none of these cases all three observers agreed, resulting in a Fleiss' kappa of −0.025.

**Table 2.** Clinical accuracy of index test using 2 x 2 tables

		Confirmatory testing^a^		Total
		Positive	Negative	
Clinical diagnosis by GP^b^	Yes	102 (74.5%)^c^	35	137
	No	0	0^d^	0
	Total	102^e^	35	137

^a^At least 1 out of 3 confirmatory tests (potassium hydroxide [KOH], polymerase chain reaction [PCR], culture) positive. ^b^At least 2 out of 3 observers positive diagnosis. ^c^ *P* value for positive predictive value (PPV) = 0.000 with 95% CI = 0.961 to 1.000. ^d^ *P* value for specificity = 0.000 with 95% CI = 0.000 to 0.036. ^e^ *P* value for sensitivity = 0.000 with 95% CI = 0.964 to 1.000.

### Sensitivity analysis of clinical diagnosis

Table 3 shows the sensitivity analysis for differences in diagnostic values between the possible combinations of index and reference tests. The index test was divided into three groups based on the number of observers confirming clinical diagnosis (1 out of 3 up to 3 out of 3). For the reference test all possible combinations were used (see Table S2–S4).

**Table 3.** Sensitivity analysis

		*n*	Sensitivity, %(95% CI)	Specificity, %(95% CI)	Positive predictive value, %(95% CI)	Negative predictive value, %(95% CI)
Clinical diagnosis	Confirmatory test					
**1 out of 3**	KOH or PCR or culture	137	100(95.5 to 100)	0(0 to 12.3)	74.5(66.2 to 81.3)	–
	KOH	139	100(95.1 to 100)	0(0 to 9.8)	67.6(59.1 to 75.2)	–
	PCR	137	100(93.8 to 100)	0(0 to 7.1)	53.3(44.6 to 61.8)	–
	Culture	68	100(87.1 to 100)	0(0 to 12.3)	48.5(36.4 to 60.9)	–
	KOH & PCR	137	100(96.6 to 100)	0(0 to 3.4)	50.4(41.7 to 59.0)	–
	KOH & culture	68	100(84.5 to 100)	0(0 to 6.7)	39.7(28.3 to 52.3)	–
	PCR & culture	66	100(80.0 to 100)	0(0 to 9.6)	30.3(19.9 to 43.0)	–
	KOH & PCR & culture	66	100(78.1 to 100)	0(0 to 9.2)	27.3(17.4 to 39.8)	–
**2 out of 3^a^**	KOH or PCR or culture	137	100(95.5 to 100)	0(0 to 12.3)	74.5(66.2 to 81.3)	–
	KOH	139	100(95.1 to 100)	0(0 to 9.8)	67.6(59.1 to 75.2)	–
	PCR	137	100(93.8 to 100)	0(0 to 7.1)	53.3(44.6 to 61.8)	–
	Culture	68	100(87.1 to 100)	0(0 to 12.3)	48.5(36.4 to 60.9)	–
	KOH & PCR	137	100(96.6 to 100)	0(0 to 3.4)	50.4(41.7 to 59.0)	–
	KOH & culture	68	100(84.5 to 100)	0(0 to 6.7)	39.7(28.3 to 52.3)	–
	PCR & culture	66	100(80.0 to 100)	0(0 to 9.6)	30.3(19.9 to 43.0)	–
	KOH & PCR & culture	66	100(78.1 to 100)	0(0 to 9.2)	27.3(17.4 to 39.8)	–
**3 out of 3**	KOH or PCR or culture	137	95.1(88.4 to 98.2)	14.3(5.4 to 31.0)	76.4(67.9 to 83.3)	50.0(20.1 to 79.9)
	KOH	139	94.7(87.5 to 98.0)	11.1(4.2 to 24.8)	69(60.2 to 76.7)	50.0(20.1 to 79.9)
	PCR	137	94.5(85.8 to 98.2)	9.4(3.9 to 19.9)	54.3(45.3 to 63.1)	60.0(27.4 to 86.3)
	Culture	68	93.9(78.4 to 98.9)	5.7(1.0 to 20.5)	48.4(35.9 to 61.2)	50.0(9.2 to 90.8)
	KOH & PCR	137	94.2(85.1 to 98.1)	8.8(3.6 to 18.9)	51.2(42.2 to 60.1)	60.0(27.4 to 86.3)
	KOH & culture	68	92.6(74.2 to 98.7)	4.9(0.8 to 17.8)	39.1(27.4 to 52.1)	50.0(9.2 to 90.8)
	PCR & culture	66	95(73.1 to 99.7)	6.5(1.7 to 18.9)	30.6(19.9 to 43.8)	75.0(21.9 to 98.7)
	KOH & PCR & culture	66	94.4(70.6 to 99.7)	6.3(1.6 to 18.2)	27.4(17.2 to 40.4)	75.0(21.9 to 98.7)

^a^Combination used for logistical regression analysis.

KOH = potassium hydroxide. PCR = polymerase chain reaction.

The diagnostic values for the first two groups were the same: a sensitivity of 100% and PPV of 27.3% to 74.5%, depending on the combination of confirmatory tests. Since all patients received a positive clinical diagnosis by at least one or two observers, specificity was 0% and NPV could not be calculated.

Compared with the first two groups, sensitivity was slightly lower for the third group (3 out of 3), ranging from 92.6%–95.1%; PPVs were roughly equal, between 27.4% and 76.4%. Specificity was 4.9%–14.3 and NPVs were between 50.0% and 75.0%.

### Logistical regression analysis

Table 4 shows the logistical regression analyses for correct clinical diagnosis for the primary combination of at least 2 out 3 observers diagnosing onychomycosis and at least 1 out of 3 confirmatory tests being positive. Univariate analysis showed male sex to increase the odds of a correct clinical diagnosis with an OR of 2.889 (95% CI = 1.233 to 6.770; *P* = 0.015).

**Table 4.** Logistical regression analysis correct diagnosis GPs

Demographic factors	Univariate analysis			Multivariate analysis		
	OR	95% CI	*P* value	OR	95% CI	*P* value
**Age, years**	1.009	0.979 to 1.041	0.548	1.066	0.983–1.157	0.124
0–25 years	Reference group		0.278	Reference group		0.175
26–50 years	4.2	0.526 to 33.543	0.176	0.965	0.040 to 23.419	0.982
51–75 years	2.522	0.335 to 18.983	0.369	0.162	0.003 to 8.986	0.374
**Male sex**	**2.889^a^**	1.233 to 6.770	**0.015^a^**	**3.873^a^**	1.230 to 12.195	**0.021^a^**
**Skin type (Fitzpatrick 4–6)**	1.03	0.104 to 10.240	0.98	0.51	0.030 to 8.581	0.64
**Clinical factors**						
**Percentage of the index toenail affected**						
1–10	Reference group		0.256	Reference group		0.218
11–25	1.818	0.312 to 10.582	0.506	5.042	0.335 to 75.802	0.242
26–50	4.182	0.741 to 23.594	0.105	10.212	0.499 to 209.180	0.132
51–75	2.778	0.472 to 16.374	0.259	2.077	0.071 to 60.829	0.671
76–100	8	0.580 to 110.268	0.12	10.66	0.143 to 794.910	0.282
**OSI score**						
Mild (0–5)	Reference group		0.117	Reference group		0.214
Moderate (6-15)	1.256	0.439 to 3.596	0.67	0.488	0.075 to 3.194	0.454
Severe (16-35)	3.077	0.908 to 10.425	0.071	2.192	0.146 to 32.856	0.57
**Total affected toenails**	0.998	0.815 to 1.223	0.986	0.962	0.714 to 1.297	0.802
**Time since onset, years**	1.028	0.980 to 1.078	0.266	1.04	0.977 to 1.107	0.217
**Time since onset >1 year**	1.048	0.348 to 3.153	0.934	0.377	0.074 to 1.926	0.241
**Score ONYCHO questionnaire**						
Good (67-100)	Reference group		0.889	Reference group		0.427
Moderate (34-66)	1.204	0.534 to 2.716	0.654	1.983	0.652 to 6.029	0.228
Poor (0–33)	0.956	0.164 to 5.556	0.96	0.919	0.091 to 9.231	0.943
**Risk factors**						
History tinea pedis	2.154	0.988 to 4.696	0.054	2.735	0.953 to 7.855	0.062
Family members with onychomycosis	0.794	0.325 to 1.940	0.613	1.3	0.363 to 4.660	0.687
Presence of a different dermatological disease	1.261	0.530 to 3.003	0.6	1.664	0.482 to 5.764	0.42
History of oral treatment	0.582	0.223 to 1.521	0.269	0.294	0.074 to 1.164	0.081
History of any previous treatment	1.719	0.658 to 4.491	0.269	**4.022^a^**	1.075 to 15.040	**0.039^a^**
History of use of immunosuppressives	0.584	0.199 to 1.719	0.329	0.55	0.128 to 2.370	0.423
Frequent use of public swimming pools, saunas or spas (≥5 visits/year)	0.923	0.416 to 2.048	0.844	1.284	0.390 to 4.229	0.682
Consistent use of flip flops during these visits	1.038	0.417 to 2.585	0.935	0.932	0.279 to 3.114	0.909
History of smoking or actively smokes	0.798	0.368 to 1.731	0.568	0.369	0.131 to 1.040	0.059

OSI = Onychomycosis Severity Index.

Statistical significance is indicated by ^a^ and bold text.

Multivariate analysis showed male sex (OR 3.873, 95% CI = 1.230 to 12.195; *P* = 0.021) and history of any previous treatment (OR 4.022, 95% CI = 1.075 to 15.040; *P* = 0.039) were significantly associated with a correct diagnosis. Stepwise forward and backward methods did not reveal any additional significant contributors.

### Subgroup analysis

Based on multivariate regression, subgroup analysis was performed for both sex and history of any previous treatment. For sex, the PPV for males was 85.0% compared with 66.2% for females. For participants with and without a history of previous treatment, this was 76.3% and 65.2%, respectively (see Table S5-6).

## Discussion

### Summary

This study demonstrated an overall accuracy of clinical diagnosis of onychomycosis by GPs of 74.5%, meaning that 25.5% of cases could not be confirmed by laboratory testing and could be considered incorrect. These patients are at risk of receiving unnecessary antifungal treatment if no confirmatory testing were performed. Male sex and a history of any previous treatment significantly increased the odds of making a correct diagnosis.

### Strengths and limitations

All data used originated from participants recruited from both general public and general practice; the observers performing clinical diagnosis were all practising GPs and did not have additional training, making the results representative for other primary care settings.

All clinical diagnoses were made independently from other observers, without the knowledge of confirmatory test results, limiting observer bias.

Although there is still debate on what should be considered the gold standard for confirmatory testing, a combination of tests is often recommended.^20,22,23,26,27^ For this study, at least one of three tests being positive was considered sufficient to minimise incorrect exclusion of true cases of onychomycosis. This is consistent with daily practice where normally a single confirmatory test is performed when in doubt.^10,18^ If one of the other recommended approaches would have been applied, clinical accuracy would have been lower, as shown in Table 3.

Regarding the sample-size calculation, a limitation was that a sensitivity of 100% was applied to the reference standard. A more conservative approach would be to take the sensitivity of 85% from the KOH test, the highest of the confirmatory tests used. Using the same calculation, the required sample size would have been a total of 132 patients.

In addition, selection bias was an inherent consequence, with all data originating from an RCT. All patients were specifically recruited for having onychomycosis. This is likely the reason that there were no cases in which a majority of observers (2 out of 3) diagnosed something other than onychomycosis. This is reflected by a specificity of 0% (Table 2), and signifies an underestimation of true clinical accuracy by GPs.

Another limitation is that clinical diagnosis in daily practice is made by a single GP instead of a group. Although sensitivity analysis showed no difference in diagnostic values between 1 out of 3 or 2 out of 3 observers, accuracy could be lower in daily practice.

Although GPs were blinded to each other’s clinical diagnosis and test results, they were aware their assessment was part of the inclusion process for the trial, potentially leading to observer bias. When in doubt, onychomycosis might have been diagnosed too easily, increasing the chance of inclusion; and vice versa, an alternative diagnosis might have been preferred, promoting milder cases to increase the chance of response to treatment. Given the incentive of inclusion, the former seems more likely and GPs are probably better in diagnosing onychomycosis in daily practice than this study suggests.

### Comparison with existing literature

Li *et al* found a clinical accuracy of 75.4%, which is consistent with the present study findings.^19^ However, clinical diagnosis primarily was made by specialists other than GPs, who were likely more experienced in diagnosing nail disease. In addition, only microscopy was used for confirmation, potentially underestimating accuracy of clinical diagnosis. Kuijpers and Tan found an accuracy of 81% for GPs.^18^ Although higher than the 74.5% found in the present study, dermatologists had an accuracy of 93%, supporting that experience improves accuracy. Using three different tests, the chance of confirming fungal infection was actually increased and therefore a correct diagnosis. Thus, an accuracy of 74.5% is arguably representative for GPs and other primary care settings.

### Implications for research and practice

In conclusion, the study demonstrates a significant chance of an incorrect clinical diagnosis without confirmatory testing in primary care, especially in female patients or patients without any previous treatment. Not performing a confirmatory test could lead to unnecessary patient exposure to antifungal treatments. The authors, therefore, would not currently recommend GPs advise therapy without confirmatory testing. With 67.9% of cases confirmed by KOH alone against relative low costs, initial KOH testing would be a reasonable approach (Table 3).

To decrease observer bias, further studies to improve validity of results should ideally include all, or at least a broader spectrum of, nail disorders and all levels of severity as far as onychomycosis is concerned. Besides reducing bias by study design, future studies could obtain additional variables that might significantly influence clinical accuracy.

Despite its limitations, this study underlines the importance of confirmatory testing, especially when considering treatment. Further research is necessary to optimise the accuracy of the clinical diagnosis by GPs and reduce the number of patients unnecessarily exposed to treatment.

## Data

The dataset relied on in this article is available from the corresponding author on reasonable request.
